# Allogeneic Cell Therapy Applications in Neonates: A Systematic Review

**DOI:** 10.1093/stcltm/szad048

**Published:** 2023-08-21

**Authors:** Abdul Razak, Donna Lei, Courtney A McDonald, Rod W Hunt, Suzanne L Miller, Atul Malhotra

**Affiliations:** Department of Paediatrics, Monash University, Melbourne, VIC, Australia; Monash Newborn, Monash Children’s Hospital, Melbourne, VIC, Australia; The Ritchie Centre, Hudson Institute of Medical Research, Melbourne, VIC, Australia; Department of Paediatrics, Monash University, Melbourne, VIC, Australia; The Ritchie Centre, Hudson Institute of Medical Research, Melbourne, VIC, Australia; Department of Obstetrics and Gynaecology, Monash University, Melbourne, VIC, Australia; Department of Paediatrics, Monash University, Melbourne, VIC, Australia; Monash Newborn, Monash Children’s Hospital, Melbourne, VIC, Australia; The Ritchie Centre, Hudson Institute of Medical Research, Melbourne, VIC, Australia; The Ritchie Centre, Hudson Institute of Medical Research, Melbourne, VIC, Australia; Department of Obstetrics and Gynaecology, Monash University, Melbourne, VIC, Australia; Department of Paediatrics, Monash University, Melbourne, VIC, Australia; Monash Newborn, Monash Children’s Hospital, Melbourne, VIC, Australia; The Ritchie Centre, Hudson Institute of Medical Research, Melbourne, VIC, Australia

**Keywords:** allogeneic, cell therapy, human amnion epithelial cell, infant, mesenchymal stromal cell, neonates, premature, preterm, total nucleated cell

## Abstract

**Background:**

Neonatal cell therapy applications are increasing; however, data on allogeneic cell therapy are limited.

**Objective:**

To summarize evidence on allogeneic cell therapy in term and preterm neonates.

**Methods:**

Cochrane Central Register of Controlled Trials, Embase, Ovid Medline, and various registries were searched for studies investigating the safety, feasibility, and efficacy of allogeneic cell therapy in neonates. Two authors independently selected the articles, extracted data, and assessed the risk of bias.

**Results:**

Twelve published (153 infants) and 21 ongoing studies were included. These studies predominantly sourced allogeneic cells from umbilical cord blood (UCB). Mesenchymal stromal cells (MSCs) were the main cell type used (134 of 153 infants); others included UCB-derived total nucleated cells (TNCs) and human amnion epithelial cells (hAECs). Applications included bronchopulmonary dysplasia (BPD; 113 infants), Krabbe disease (13 infants), intraventricular haemorrhage (10 infants), perinatal arterial ischemic stroke (10 infants), hypoxic-ischaemic encephalopathy (6 infants), and necrotizing enterocolitis (1 infant). Nine out of 12 studies did not report any serious adverse events (SAEs) related to cell administration. Three studies reported SAEs, such as graft versus host disease (GVHD) in 5 infants (UCB-derived TNCs for Krabbe disease); and transient cardiorespiratory compromise in 1 infant (hAECs for BPD). Data on efficacy outcomes were limited.

**Conclusion:**

The safety and feasibility of allogeneic cell therapy applications in neonates are available, mainly from the use of MSCs. Further safety data for other cell types are required, and the risk of GVHD in different settings needs to be determined. Efficacy studies are largely lacking for all cell types.

**Protocol Registration:**

The protocol was registered with PROSPERO (registration number CRD42023397876), the international prospective register for systematic reviews (https://www.crd.york.ac.uk/PROSPERO).

## Graphical Abstract

**Graphical Abstract:**
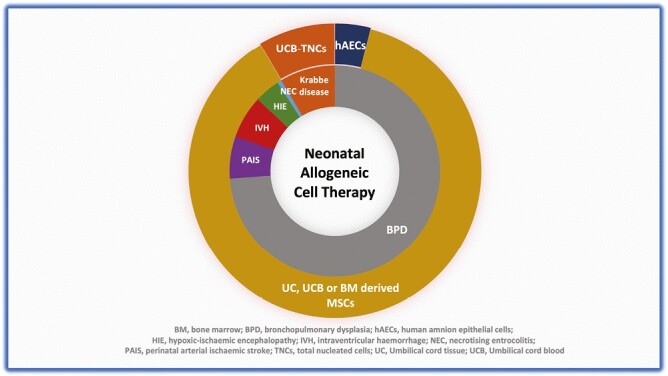

**Significance Statement**

The present study systematically summarizes the evidence on using all types of allogeneic cells relevant to the neonatal period in term and preterm infants from published and ongoing studies. The results show some evidence of the safety and feasibility of certain types of mesenchymal cells and highlight the lack of efficacy data for all the cell types. These findings suggest further research on a broader population of allogeneic cells is required, particularly emphasizing their efficacy related to the predominant morbidities that require therapeutic intervention in the neonatal period.

## Introduction

Cell therapy is a potential paradigm shift in neonatal medicine. In particular, it is a promising therapy to reduce the complications of prematurity and perinatal asphyxia.^1,2^ It involves administering biological living cells to prevent or reverse the disease process and normalize the structure and function of organs and tissues by reducing inflammation and promoting endogenous repair of damaged or diseased cells or tissues.^3^ Cell therapy is commonly used to treat cancers and hematological disorders, but its use in neonates for regenerative and immunomodulatory applications remains a relatively new field with many unanswered questions.^4,5^

Preclinical studies have demonstrated the potential role of cell therapies to prevent or treat pathology associated with prematurity, including bronchopulmonary dysplasia (BPD),^6^ preterm brain injury,^7^ necrotizing enterocolitis (NEC),^8^ and other conditions, such as perinatal asphyxia,^9^ perinatal arterial ischaemic stroke (PAIS),^10^ and congenital heart disease.^11^ These preclinical studies demonstrate that, depending on the cell type and timing of administration, cell therapy may modulate tissue injury via an anti-inflammatory,^12-14^ anti-apoptotic,^15^ paracrine,^16^ or angiogenic effects^17^ or, in the case of some stem cells, may replace damaged cells.^18^ These benefits are also shown in clinical studies investigating the role of cell therapy in several neonatal diseases, with a few studies showing that it could be promising^19,20^; however, the data are not robust.^21^

Cell therapies are broadly categorized as autologous (using one’s own cells) or allogeneic (cells from others). While it is unknown whether there are differences between these 2 types of cell therapy, research is ongoing to examine the safety and efficacy of both types of cell therapies in various neonatal conditions. Research on allogeneic cell therapy has been gaining momentum,^22,23^ given that autologous cell therapy is not feasible for many infants, as complicated or preterm births are not usually anticipated, and in some babies, the target cell dose required may not be achievable.^24^

However, allogeneic cell therapy is not without challenges. Allogeneic cell therapies are expensive, and some require extensive logistical support. As cells in allogeneic cell products are obtained from healthy donors, there is a risk of immunological reactions, such as graft-versus-host disease (GVHD). Furthermore, due to the recognition of donor cells by the immune system, donor cells can be rapidly removed from the system before they can offer any therapeutic or regenerative benefits. Hence, it is important to understand whether allogeneic cell therapy in the neonatal period is safe, feasible, and efficacious. The aim of this systematic review was to examine the published evidence that currently exists regarding the safety, feasibility, and efficacy of allogeneic cell therapy applications for newborn infants.

## Methods

The systematic review was conducted as per Preferred Reporting Items for Systematic Reviews and Meta-analyses reporting guideline. The study protocol was registered with PROSPERO (registration number CRD42023397876), the international prospective register for systematic reviews (https://www.crd.york.ac.uk/PROSPERO).

### Search Strategy

Literature search was conducted using prespecified search terms within the following databases: Cochrane Central Register of Controlled Trials (CENTRAL), Embase, and Ovid Medline from inception through October 2022 without any language restriction. In addition, reference lists and citations of included studies and relevant reviews were also searched. Finally, the following clinical trial registries: Australian New Zealand Clinical Trials Registry (https://www.anzctr.org.au/TrialSearch.aspx), Chinese Clinical Trial Registry (www.chictr.org.cn), European Clinical Trials Registry (https://www.clinicaltrialsregister.eu), International Clinical Trials Registry Platform (https://www.who.int/clinical-trials-registry-platform), and International Standard Randomised Controlled Trial Number Registry (https://www.isrctn.com), and U.S. National Library of Medicine (https://clinicaltrials.gov) were also explored. Search strategy details across each database are provided in the Supplementary Material.

### Eligibility Criteria

#### Type of Studies

All published and ongoing human clinical studies using allogeneic cells in neonates with or without a control arm and evaluating the feasibility, safety, or efficacy were included. Allogeneic cells were considered as biological living cells with broad or specific regenerative or immunomodulatory potential obtained from a donor and intended for administration into a genetically distinct related or unrelated recipient.

#### Population

Term or preterm neonates with or without neonatal morbidities.

#### Interventions

Studies using allogeneic cell therapy for any neonatal condition, irrespective of source, route of administration, and dosing, during the first 4 weeks of life in full-term neonates or until 4 weeks of corrected age (44 weeks’ postmenstrual age) in preterm neonates were included.

#### Outcomes

The following outcomes were evaluated:

1. Safety, including any issues related to infusion or any complications, such as infection, anaphylaxis, and GVHD, as defined by authors.
2. Feasibility, including the ability to carry out therapy once eligibility is confirmed, as defined by authors.
3. Clinical outcomes, as defined by authors, included death, neurodevelopmental impairment, cerebral palsy, brain injury (intraventricular haemorrhage (IVH), white matter injury, or seizures), BPD, retinopathy of prematurity, NEC, and a composite of death or any morbidities.

### Study Selection, Data Abstraction, and Assessment of the Methodological Quality

A.R. and A.M. independently read the titles and abstracts of the final list of records assimilated using Covidence systematic review software, Veritas Health Innovation, Melbourne, Australia, available at www.covidence.org. They also independently reviewed the shortlisted full-text articles for inclusion. Furthermore, A.R. and D.L. independently extracted the relevant information from included studies. In addition, A.R. and D.L. independently assessed the methodological quality using the Cochrane Risk of Bias tool (version 2) for randomized studies,^25^ modified New Ottawa Scale for non-randomized studies,^26^ and another tool for studies with no comparator group.^27^ Finally, discrepancies were resolved by discussion and consensus.

## Results

**Figure 1.**
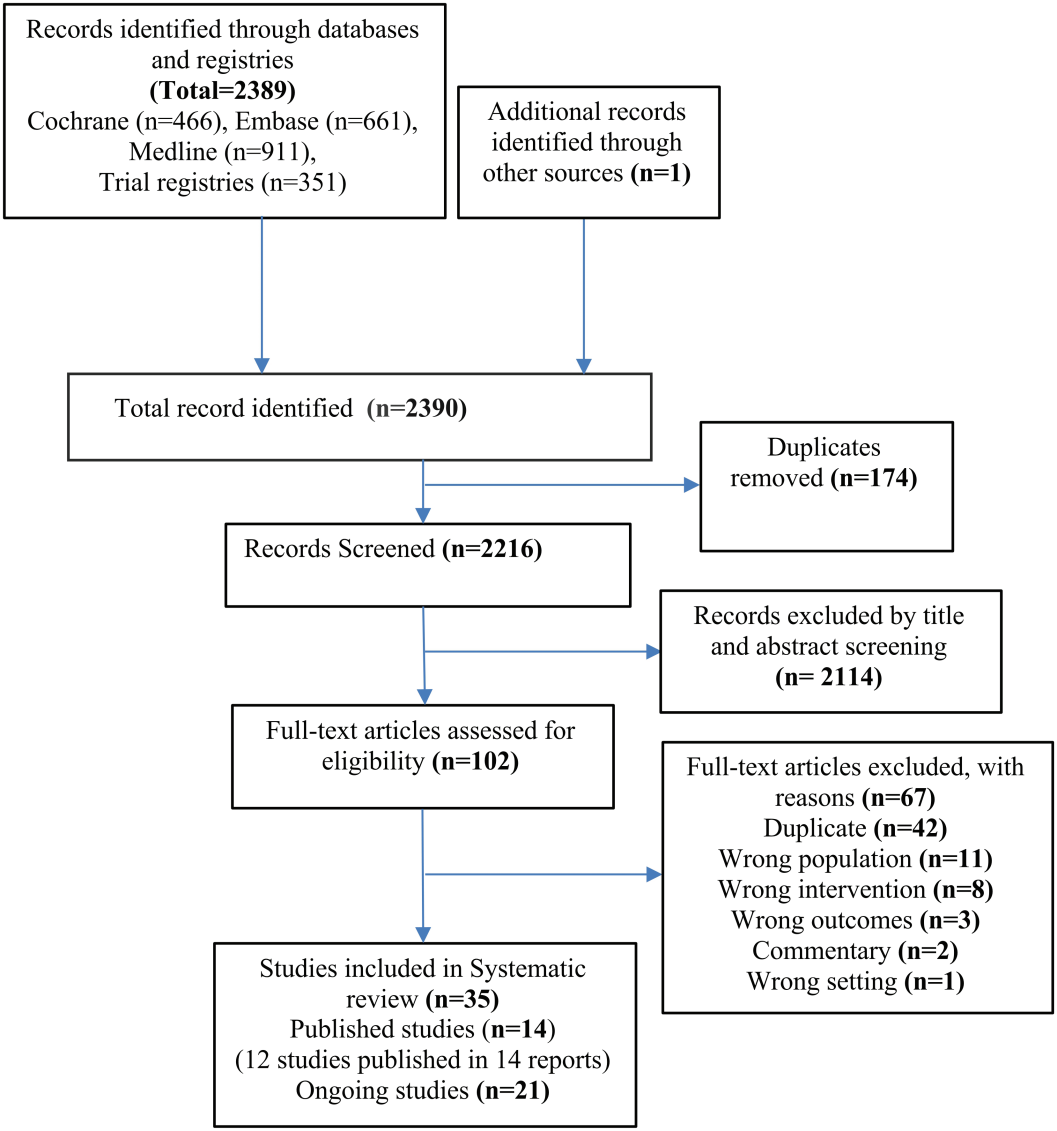
Study flow diagram outlining stages of search results and filtering process (as per Preferred Reporting Items for Systematic Reviews and Meta-Analyses (PRISMA) guidelines).

The systematic search yielded 2390 records. After excluding 174 duplicates, 2216 articles were screened, and 102 articles were selected for full-text reading. Furthermore, 67 articles were excluded for the reasons mentioned in the study selection log (Fig. 1). Twelve studies (published as 14 reports)^20,28-40^ and 21 ongoing studies^41-63^ were included. One study published its findings in 2 reports, 1 assessing short-term outcomes^35^ and the other assessing long-term^28^ outcomes. Similarly, another study reported results at different follow-up periods as 2 reports.^31,37^

### Published Studies

Details of included studies are summarized in Table 1. Of the 12 studies, 5 were phase I studies with no control arm,^29,33,36,39,40^ 3 were case reports,^30,34,38^ and the remainder were phase II randomized controlled trial,^20^ cohort study (phase I clinical trial with historic case-matched controls),^28,35^ descriptive study,^31,37^ and a case series.^32^ These studies included 153 term and preterm infants worldwide who received allogeneic cell therapy in the neonatal period.

**Table 1.** Characteristics of included studies.

Study	Location	Design	Population, sample size^*^	Intervention	Comparator	Safety and feasibility	Clinical outcomes
Neurological conditions (6 studies, 39 participants)							
Ahn et al^29^	South Korea	Open-label, phase I dose-escalation trial without control arm	Infants 24-30 weeks with severe IVH *n* = 9	Intraventricular injection of allogeneic UCB-MSCs 5 × 10^6^ − 1 × 10^7^/kg single-dose days 7-15	N/A	Safety: all infants tolerated the injection well, with no immediate complications or death within 6 hours after transplantation 88.9% (8/9) infants developed serious adverse events related to prematurity Feasibility: Authors deemed the therapy as feasible	Death: all discharged alive BPD: 88.9% (8/9) developed BPD, with 44.4% (4/9) of severe severity Brain injury: 11.1% (1/9) developed seizures after injection NEC: 22.2% (2/9) developed NEC ROP: 22.2% (2/9) developed ROP requiring surgery
Allewelt et al (1996-2010)^31^ and Escolar et al (1998-2004)^37^	US	Descriptive study (retrospective)	Infants with early infantile Krabbe disease Allewelt*, n* = 12 (1996-2010) Escolar, *n* = 7 (1998-2004)	Allogeneic UCB-total nucleated cells with a dose of ≥3 × 10^7^/kg day 12-28 with at least 4/6 HLA matching	N/A	Safety: 57% (4/7) infants developed acute GVHD (provided in Escolar et al) Feasibility: N/A (authors stated that they evaluated feasibility, however, did not include a final assessment)	Death: 0.0% (0/7) at 36 months in Escolar et al study; 16.6% (2/12) died by 5 years from transplant complications in Allewelt et al study Brain injury: 100% (7/7) normal progression of myelination on MRI in Escolar et al (3-years follow-up); At 5 years, 80.0% (8/10) had spasticity, 0.0% (0/10) had seizures, and none had vision or hearing loss in Allewelt et al
Baak et al^33^	Netherlands	Open-label, phase I trial without control arm	Infants ≥ 36 weeks with suspected perinatal arterial ischaemic stroke *n* = 10	One dose of intranasal allogeneic BM-MSCs 45-50 × 10^6^ within 7 days of stroke (day 12-29)	N/A	Safety: No serious adverse events or signs of toxicity detected for 3 months after MSC administration (one had fever, 38^o^ C, 1h after administration, which resolved spontaneously within 2h) Feasibility: 100% (10/10) infants, able to diagnose and treat within 7 days of presenting with signs of PAIS	Death: 100% (10/10) infants alive at 3 months Brain injury: 0% (0/10) infants had unexpected structural cerebral abnormalities at 3 months Early neurological abnormality: 30% (3/10) had abnormal GMA/HINE examination
Bozkaya et al^34^	Turkey	Case report	Preterm infant 27 weeks with severe IVH *n* = 1	1 each of dose of Intraventricular and intravenous allogeneic UCB-MSCs 1 × 10^7^/kg day 6	N/A	Safety: Infant alive at 2 years Feasibility: N/A	Death: Infant alive at 2 years Brain injury: At day 133, MRI showed normal ventricles and hemosiderin accumulation in the parenchyma; normal neurological outcome at 2 years of age
Cotten et al^36^	US	Open-label, phase I without control arm	Infants 36-41 weeks with HIE *n* = 6	Intravenous allogeneic UC-MSCs 2 × 10^6^/kg at 48 hours and 2 months (one infant received only 1 dose)	N/A	Safety: No infants experienced infusion reactions and all infant blood cultures were negative Feasibility: N/A	Death: All infants survived at 6 months
Lesnik et al^38^	US	Case report	Term infant 38 weeks with Krabbe’s disease *n* = 1	Allogeneic UCB cells day 19 (type, route, and dose information not provided)	N/A	Safety: Infant developed grade III GVHD Feasibility: N/A	Death: Infant alive at 8 months of age
Pulmonary conditions (5 studies, 113 participants)							
Chang et al^35^ and Ahn et al^28^	South Korea	Cohort study (Open-label, phase I with matched controls)	Preterm infants 23-29 weeks at risk of BPD *n* = 27	Intratracheal allogeneic UCB-MSCs 1 × 10^7^/kg for first 3 infants and 2 × 10^7^/kg for next 6 infants days 7-14	Historical control group with no UCB-MSCs and matched for gestational age, birth weight, and respiratory severity (2:1 ratio)	Safety: No immediate serious adverse events or dose-limiting toxicity; no difference in incidence of serious adverse events between MSCs and comparison group within 84 days after administration Feasibility: Authors deemed the therapy as feasible	Death: Within 2 years corrected age 0.0% (0/18) control vs. 11.1% (1/9) MSCs, died after discharge from sepsis BPD: Severity, control vs. MSCs, mild 27.8% (5/18) vs. 66.7% (6/9); moderate 27.8% (5/18) vs. 33.3% (3/9); severe 44.4% (8/18) vs. 0/0% (0/9), *P* = .037 Brain injury, control vs. MSCs: IVH grade ≥ 3 0.0% (0/18) vs. 0.0% (0/9); PVL 6% (1/18) vs. 11% (1/9) NEC: stage ≥ 2b 11.1% (2/18) control vs. 11.1% (1/9) MSCs, *P* = 1.0 ROP: Grade ≥ 3 50.0% (9/18) control vs. 11.1% (1/9) MSCs, *P* = .26 Brain injury: Cerebral palsy 7.1% (1/14) control group vs. 0.0% (0/8) MSCs group, *P* = 1.00; Low MDI 10% (1/10) control group vs. 0% (0/8) MSCs group; No difference in blindness (0% vs. 0%) and hearing loss (0% vs. 0%)
Ahn et al^20^	South Korea	Phase II randomized controlled trial	Preterm infants 23-28 weeks at risk of BPD *n* = 66	Intratracheal allogeneic UCB-MSCs 1 × 10^7^/kg days 5-14	Intratracheal normal saline of equal volume	Safety: No serious adverse events related to MSCs identified Feasibility: Authors deemed the therapy as feasible	Death: 3.0% (1/33) control vs. 9.1% (3/33) MSCs, *P* = .30 BPD: Severe BPD 42.4% (14/33) control vs. 27.3% (9/33) MSCs, *P* = .20 (no difference in mild or moderate BPD also; subgroup analysis shows lower severe BPD in 23-24 weeks GA subgroup (19% (3/16) vs. 53% (8/15); *P* = .04) NEC: stage ≥2b 15.2% (5/33) control vs. 9.1% (3/33) MSCs, *P *= .45 ROP: Grade ≥ 3 39.4% (13/33) control vs. 23.3% (7/30) MSCs, *P *= .17
Alvarez-Fuente et al^32^	Spain	Case series	Preterm infants 24 weeks with severe BPD *n* = 2	Intravenous allogeneic BM-MSCs Patient 1: Increasing weekly dose for 5 weeks, 1.1-13.9 × 10^6^/kg at 5 months (exact postnatal age not available) Patient 2: For 3 weeks, 5 × 10^6^/kg day 85	N/A	Safety: No acute adverse events related to MSCs in both patients Feasibility: Authors deemed the therapy as feasible	Death: 100% (2/2) infants died, from pneumothorax and respiratory deterioration BPD: No evident respiratory improvement in patient 1, variable changes in lung injury biomarkers in both patients
Lim et al^39^	Australia	Open-label, phase I trial without control arm	Preterm infants 24-28 weeks with BPD *n* = 6	Intravenous allogeneic hAECs 1 × 10^6^/kg days 59-187	N/A	Safety: 16.7% (1/6) infants had transient cardiorespiratory compromise during cell administration (pulmonary embolism), no similar events in other babies after inline filter Feasibility: Authors deemed the therapy as feasible	Death: 16.7% (1/6), 1 month after hAEC administration due to multiorgan failure unrelated to hAECs BPD progression: 100% (5/5) surviving infants had complications of BPD (systemic or pulmonary hypertension) (authors report other postnatal morbidities, such as IVH, ROP, NEC but they occurred before hAECs)
Powell et al^40^	US	Open label, phase I dose-escalation trial without control arm	Preterm stable infants 23-28 weeks and birth weight 500-1000 g requiring mechanical ventilation with no surfactant within 24 hours *n *= 12	Intratracheal UCB-mesenchymal stromal cells 1 10^7^/kg (*n* = 6) and 2 × 10^7^/kg (*n* = 6) day 5-14	N/A	Safety: No significant cardiorespiratory decompensation within 6 hours; no dose-limiting toxicities within 72 hours; 66.7% (8/12) infants developed serious adverse events but none related to the study drug Feasibility: Authors deemed the therapy as feasible but no definition provided	Death: 8.3% (1/12) infants died from pulmonary hypertension following completion of study BPD: 83.3% (10/12) infants had severe BPD and 16.6% (2/12) had mild BPD Brain injury: 0% (0/12) infants developed severe IVH NEC: 0% (0/12) infants developed NEC ROP: 91.7% (11/12) infants developed ROP
Gastrointestinal conditions (1 study, 1 participant)							
Akduman et al^30^	Turkey	Case report	Term infant 37 weeks with SVT-related NEC *n* = 1	Intravenous allogeneic UC-MSCs 1 × 10^7^ day 26	N/A	Safety: No adverse events mentioned Feasibility: N/A	Death: None NEC Progression: Increased intestinal blood flow following MSCs

^*^We included data from eligible infants only.

Abbreviations: GMA: general movement assessment; GVHD: graft versus host disease; hAECs: human amnion epithelial cells; HIE: hypoxic-ischaemic encephalopathy; HINE: Hammersmith infant neurological examination; IVH: intraventricular haemorrhage; MRD-BMT: matched-related donor bone marrow transplantation; MRI: magnetic resonance imaging; MSC: mesenchymal stem cells; N/A: not applicable; NEC: enterocolitis; PAIS: arterial ischaemic stroke. PDA: patent ductus arteriosus; PVL: periventricular leukomalacia; RBC: red blood cell; ROP: retinopathy of prematurity; SVT: supraventricular tachycardia; UC: umbilical cord; UCB: umbilical cord blood; UCBT: umbilical cord blood transplantation.

#### Risk of Bias Assessment of Included Studies

Methodological assessment for each study is provided in the Supplementary material. A complete methodological evaluation was not performed for one report where only the abstract was available.^36^ Nine studies with no control arm (phase I studies, descriptive study, case report, and case series) scored between 3/8 and 6/8.^29-34,37-40^ Five of these 9 studies were deemed at low-risk,^29,31,33,37,39,40^ and the remainder were at moderate (3 studies)^30,32,38^ and high (1 study)^34^ risk of bias. The phase II randomized trial^20^ and the cohort study^28,35^ were at low risk of bias.

#### Allogeneic Cells: Indication, Source, Dose, and Route

**Figure 2.**
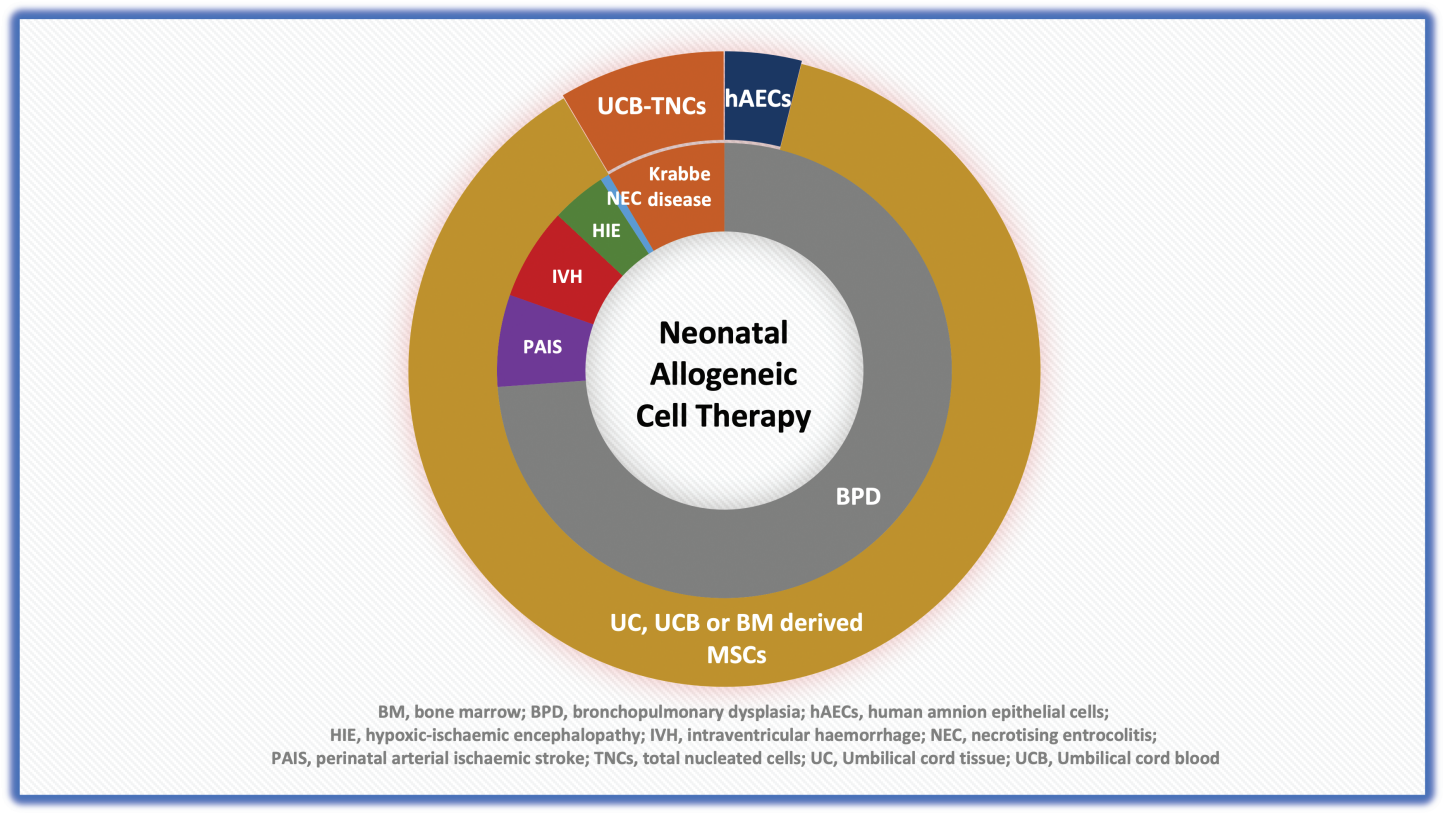
Types of allogeneic cells used and their indications in the neonatal period.

Allogeneic cells were used in human neonatal trials for various disease conditions, most commonly being BPD (5 studies, 113 infants) (Fig. 2). Other conditions include Krabbe disease (2 studies, 13 infants),^31,37^ IVH (2 studies, 10 preterm infants),^29,34^ PAIS (1 study, 10 infants),^33^ hypoxic-ischaemic encephalopathy (HIE) (1 study, 6 infants),^36^ and NEC (1 report, 1 infant).^30^ Most studies used mesenchymal stromal cells (MSCs) (134 of 153 infants in 9 studies) derived from UCB (5 studies),^20,28,29,34,35,40^ umbilical cord tissue (2 studies),^30,36^ and bone marrow (2 studies),^32,33^ with a cumulative dose between 2 and 20 million MSCs per kg^20,28,29,32,34,35,40^ or 45 to 50 million cells total.^33^ Other studies used UCB-derived total nucleated cells (TNCs)^31,37^ of 30 million per kg of body weight and human amnion epithelial cells (hAECs)^39^ of 1 million per kg of body weight. The intravenous route was commonly used^30-32,36-39^; however, a few studies administered cells via intraventricular,^29,34^ intranasal,^33^ and intratracheal routes.^20,28,35,40^ In one report, cells were administered via intravenous and intraventricular routes.^34^

#### Neurological Conditions (6 Studies)

##### Krabbe Disease

Allewelt et al (1996-2010, *n* = 12)^31^ and Escolar et al (1998-2004, *n* = 7)^37^ summarized outcomes of infants who received partial HLA-matched UCB-derived TNCs within 28 days of life from the same center. Allewelt et al reported follow-up findings until 3 years of age,^31^ whereas Escolar et al reported findings until 5 years.^37^ In both reports, the authors mentioned a favorable natural history of disease and neurological outcomes in infants who received cell therapy in the neonatal period compared with the infants who received cell therapy in the postneonatal period. However, Escolar et al reported that among newborn infants, 57% (4/7) experienced acute GVHD of grade I-II severity, while 42% (3/7) developed chronic GVHD.^37^ Furthermore, Allewelt et al reported death in 16.6% (2/12) and spasticity in 80% (8/10) infants at 5 years.^31^ In another report on Krabbe disease, Lesnik et al reported a term infant who underwent allogeneic cell therapy and developed GVHD and steroid-induced hypertrophic cardiomyopathy, which were managed appropriately with medical therapies with complete resolution at 8 months.^38^ It is important to note that the purpose of cell therapy in these reports differs from rest of other reports in that the intended therapeutic approach is transplantation. In these reports, transplantation procedure was preceded by myeloablative conditioning and followed by post-transplant immune suppression for prophylaxis against GVHD.

##### IVH

One study reported that intraventricular injection of allogeneic UCB-MSCs at a dose of 5 to 10 million cells per kg in preterm infants with severe IVH was safe, as demonstrated by the absence of immediate serious adverse events and lack of dose-limiting toxicity.^29^ In addition, none of the infants died or had serious adverse events related to the therapy. The authors also deemed the treatment feasible. In another report by Bozkaya et al, a preterm infant had complete resolution of severe IVH with a normal neurological outcome and no deficits at 2 years following a dose of intraventricular and intravenous allogeneic UCB-MSCs in the neonatal period.^34^

Other neurological disorders: Baak et al utilized a single dose of intranasal allogeneic BM-MSCs for 10 infants with PAIS within 7 days of diagnosis and showed it as safe and feasible with all infants surviving at 3 months and without any unexpected cerebral abnormalities on magnetic resonance imaging.^33^ Similarly, another report by Cotten et al shows the safety of intravenous allogeneic umbilical cord tissue-derived MSCs in infants with perinatal HIE, with all infants surviving at 6 months.^36^ However, the authors have yet to report the long-term neurodevelopmental outcomes in these infants at 12 to 16 months of age.

#### Pulmonary Conditions (5 Studies)

Three studies used intratracheal UCB-MSCs,^20,28,35,40^ one study used intravenous bone marrow-MSCs,^32^ and another used intravenous hAECs^39^ for infants at risk of BPD or with BPD. The study using intravenous hAECs in extremely preterm infants with BPD showed it as safe and feasible, but one infant had transient cardiorespiratory compromise during cell administration secondary to presumed pulmonary embolism,^39^ which did not occur in other babies after the change of protocol, including use of an inline filter and infusion over a longer period of time. Furthermore, a study by Powell et al reported intratracheal administration of UCB-MSCs as safe and feasible in 12 extremely preterm infants requiring mechanical ventilation with no serious adverse events related to the therapy.^40^ Similarly, another study by Chang et al reported the safety and feasibility of intratracheal UCB-MSCs in extremely preterm infants.^35^ The authors also compared treated infants with matched historical controls and noted no differences in clinical outcomes, except for differences in the severity of BPD between the groups. Intratracheal UCB-MSCs administration was associated with higher mild BPD but lower severe BPD. In addition, the authors provide neurodevelopmental follow-up data in another report, which showed no differences in clinical outcomes.^28^ Finally, one phase II randomized controlled trial (RCT) comparing intratracheal UCB-MSCs versus placebo found no difference in clinical outcomes, including BPD. But, in the 23-24 weeks GA subgroup, lower severe BPD was found in the intratracheal UCB-MSCs group.^20^

### Registered or Ongoing Studies

Twenty-one ongoing studies were included evaluating allogeneic cell therapy in 1020 term and preterm infants.^41-63^ The majority of the studies are using MSCs (16 studies; 1 to 80 million cells per kg)^41,44-59,62,63^; however, a few are using hAECs (1 study; 2-10 million cells per kg)^60^ and UCB-derived mononuclear cells (1 study; 0.5-3 million cells per kg),^61^ neural progenitor cells (1 study; 12 million cells per kg),^42^ and CL2020 cell, a multilineage-differentiating stress-enduring cell product (1 study; 1.5 to 15 million cells per kg)).^43^

Most studies (17 studies, 833 participants) are assessing the role of allogeneic cell therapy in extremely preterm infants with or at risk of BPD,^44-58,60-63^ with the majority being phase I trials evaluating safety and feasibility; however, a few randomized trials (6 RCTs with a sample size between 57 and 200) have been planned to determine the efficacy of clinical outcomes. In addition, a few studies (3 studies, 151 participants) are assessing allogeneic cell therapy in neurological conditions, with 2 trials focusing on HIE (neural progenitor cells and CL2020 cells)^42,43^ and one trial on IVH (MSCs).^41^

Few studies (19%) have been registered for nearly a decade but are yet to be completed.^42,50,53,62^ Furthermore, the recruitment status of many studies (43%) is unknown.^41,42,47-51,56-58^ Finally, few studies (14%) have been completed, but reports are yet to be published.^53,61,63^ A summary of all the ongoing trials on neonatal allogeneic cell therapy is provided in Table 2.

**Table 2.** Characteristics of ongoing studies.

Study	Status	Location	Design	Population	Intervention	Comparator	Primary outcome of the study
Neurological (3 studies, 151 participants)							
Efficacy and safety of pneumostem for IVH in premature infants (phase IIa) NCT02890953^41^	Unknown (2016)	South Korea	RCT	23-34 weeks with IVH grade 3-4 *n* = 22	Intracerebroventricular injection of UC-MSCs via ventricular tap (dose and timing information not provided)	Intracerebroventricular injection of normal saline via ventricular tap	Death or shunt operation until 40 weeks corrected age
Neural progenitor cell and paracrine factors to treat hypoxic ischemic encephalopathy (NCT02854579)^42^	Unknown (2013)	China	RCT	≥34 weeks and weight ≥ 2000 g with signs of encephalopathy *n* = 120	Intrathecal neural progenitor cell 4 × 10^6^ days 2-3, 5, and 10 Intrathecal concentrated paracrine factors of human MSCs 0.5 mL at 12, 24, and 48 hours Both intrathecal neural progenitor cell and intrathecal paracrine factors of human MSCs	Routine therapy	Safety until 7 days and efficacy (neonatal behavior assessment) at 2 and 4 weeks
The clinical trial of CL2020 cells for neonatal hypoxic ischaemic encephalopathy (SHIELD) (NCT04261335)^43^	Ongoing (2020)	Japan	Phase I dose-escalation trial without control arm	>36 weeks and weight > 1800g with encephalopathy *n* = 9	Intravenous CL2020 1.5 or 15 × 10^6^ day 5-14	N/A	Adverse events at 12 weeks
Pulmonary (17 studies, 833 participants)							
Intratracheal umbilical cord-derived mesenchymal stem cell for the treatment of bronchopulmonary dysplasia (NCT03645525)^44^	Ongoing (2018)	China	RCT	Extremely preterm infants with BPD *n* = 180	Intratracheal human UC-MSCs 2 × 10^7^/kg	Normal saline	Oxygen requirement at 3 days post-transplantation
Human amnion cells for the prevention of bronchopulmonary dysplasia: a protocol for a phase I dose escalation study (ACTRN12618000920291)^60^	Ongoing (2018)	Australia	Phase I dose-escalation trial without control arm	<29 weeks at high risk of severe BPD *n* = 24	Intravenous hAECs 2-10 × 10^6^/kg day 14-18	N/A	Safety until 2 years corrected age
Allogeneic human umbilical cord-derived mesenchymal stem cells for severe bronchopulmonary dysplasia in children: study protocol for a randomized controlled trial (NCT01775774)^45^	Ongoing (2019)	China	RCT	0-1 years with severe BPD *n* = 72	Intravenous human UC-MSCs 2.5-5 × 10^6^/kg (timing information not provided)	Routine supportive treatments for BPD	Cumulative duration of oxygen therapy
Clinical trial: Feasibility and security of the treatment of bronchopulmonary dysplasia in preterm babies with expanded umbilical cord allogeneic fetal mesenchymal stem cells (EudraCT 2014-003108-56)^62^	Ongoing/restarted (2014)	Spain	Phase I trial with historical controls	<28 weeks or weight < 1250 g who are candidates for corticoid therapy *n* = 10	Intravenous UC-MSCs 5 mL/kg, 3 doses (timing information not provided)	Historical controls	Feasibility and security of MSC therapy
Clinical controlled study of allogeneic cord blood in the prevention and treatment of bronchopulmonary dysplasia in premature infants (ChiCTR2000035227)^63^	Recruitment completed (2017)	China	Non-randomized observational study	<28 weeks and weight < 1250g at high risk of BPD *n* = 24	Umbilical cord blood transfusion (dose and timing information not provided)	Normal saline	Duration of oxygen therapy and invasive and non-invasive mechanical ventilation
Safety of allogeneic human cord blood-derived mononuclear cells for extreme preterms infants at high risk of death: a descriptive study^61^	Completed but not peer-reviewed or published (2015)	China	Phase I single arm study	<28 weeks requiring long-term invasive respiratory support	UCB mononuclear cells 0.5-3 × 10^6^/kg	N/A	Safety until 48 months of corrected age
Cellular therapy for extreme preterm infants at risk of developing bronchopulmonary dysplasia (NCT04255147)^46^	Not yet recruiting (2022)	Canada	Phase I dose-escalation trial without control arm	<28 weeks intubated on mechanical ventilation *n* = 9	Intravenous allogeneic UC-derived mesenchymal stromal cells 1-10 × 10^6^/kg day 7-21	N/A	Dose-limiting toxicity
Human mesenchymal stem cells for bronchopulmonary dysplasia (NCT03558334)^47^ Follow-up study of mesenchymal stem cells for bronchopulmonary dysplasia (NCT03873506)^48^	Unknown (2018)	China	Phase I non-randomized dose-escalation trial	Preterm infants with BPD *n* = 12 (follow-up study, *n* = 30)	Intravenous UC-MSCs 1-5 × 10^6^/kg (timing information not provided)	No UC-MSCs	Adverse reactions within 24 hours Follow-up study: readmission rate and length of hospital stay due to respiratory infection within 2 years
Human mesenchymal stem cells for infants at high risk for bronchopulmonary dysplasia (NCT03774537)^49^	Unknown (2019)	China	Phase I non-randomized dose-escalation trial	23-28 weeks and weight 500-1000 g intubated on mechanical ventilation *n* = 20	Intravenous UC-MSCs 1-5 × 10^6^/kg day 5-14	No UC-MSCs	Adverse reactions within 24 hours
Intratracheal umbilical cord-derived mesenchymal stem cells for severe bronchopulmonary dysplasia (NCT01207869)^50^	Unknown (2010)	China	RCT	Extremely preterm infants with severe BPD *n* = 10	Intratracheal UC-MSCs 3 × 10^6^/kg (timing information not provided)	Normal saline	Cytokine concentrations in BAL fluid and PAP at 20 weeks
Stem cells for bronchopulmonary dysplasia (NCT03378063)^51^	Unknown (2017)	China	Cohort study	Preterm infants with BPD *n* = 100	UC-MSCs (route, dose and timing information not provided)	No UC-MSCs	Death within 2 years
Mesenchymal stem cells for prevention of bronchopulmonary dysplasia in infants (NCT03631420)^52^	Ongoing (2018)	China	Phase I dose-escalation trial without control arm	23-29 weeks and weight 501-1249 g with BPD *n* = 9	UC-MSCs 3-30 × 10^6^/kg (route and timing information not provided)	N/A	Adverse events at 3 months
Long-term safety and efficacy follow-up study of PNEUMOSTEM in patients who completed PNEUMOSTEM phase-I study (NCT02023788)^53^	Recruitment completed (2014)	South Korea	Phase I trial without control arm	23-29 weeks at risk of BPD *n* = 8	Intratracheal UC-MSCs 1-2 × 10^7^/kg days 7-14	N/A	Adverse reactions at 60 months corrected age
PNEUMOSTEM for the prevention and treatment of severe BPD in premature infants (NCT03392467)^54^ Follow-up study of safety and efficacy in subjects who completed PNEUMOSTEM phase II (MP-CR-012) clinical trial (NCT04003857)^55^	Ongoing (2019)	South Korea	Phase II RCT	23-25 weeks and weight 500-1250g using ventilator *n* = 60	Intratracheal UC-MSCs 1 × 10^7^/kg day 5-14	Intratracheal normal saline	Severe BPD or death at 36 weeks postmenstrual age Safety and efficacy at 60 months corrected age
The treatment of bronchopulmonary dysplasia by intratracheal instillation of mesenchymal stem cells (NCT03683953)^56^	Unknown (2018)	China	RCT	28-37 weeks using ventilator *n* = 200	Intratracheal MSCs 25 × 10^6^/kg day 14	Intratracheal normal saline day 14	Patients with BPD at 28 days
Umbilical cord mesenchymal stem cells transplantation in the treatment of bronchopulmonary dysplasia (NCT04062136)^57^	Unknown (2019)	Vietnam	Phase I trial without control arm	1-6 month old infants with BPD *n* = 10	Intravenous UC-MSCs 1 × 10^6^/kg at baseline and 1 week after	N/A	Adverse events and patients not on O2 at 9 months
Human mesenchymal stem cells for moderate and severe bronchopulmonary dysplasia (NCT03601416)^58^	Unknown (2019)	China	RCT	Infants with BPD *n* = 57	Intravenous UC-MSCs 1 or 5 × 10^6^/kg (timing information not provided)	No UC-MSCs	Duration of O2 therapy
Cardiac (1 study, 36 participants)							
Mesenchymal stromal cells for infants with congenital heart disease (MedCaP) (NCT04236479)^59^	Ongoing (2020)	US	Phase I dose-escalation trial without control arm	Infants ≤ 3 months with repair for congenital heart defects *n* = 36	BM-mesenchymal stromal cells through cardiopulmonary bypass 1-80 × 10^6^/kg (timing information not provided)	N/A	Adverse events and/or early treatment discontinuations at 45 days

Abbreviations: BAL: bronchoscopy and bronchoalveolar lavage; BM: bone marrow; BPD: bronchopulmonary dysplasia; hAECs: human amnion epithelial cells; hCT-MSC: human umbilical cord tissue-derived mesenchymal stromal cells; HLSC: human liver stem cells; IVH: intraventricular haemorrhage; MSCs: mesenchymal stromal cells; PAP: pulmonary alveolar proteinosis; RBC: red blood cell; RCT: randomized clinical trial; ROP: retinopathy of prematurity; UC: umbilical cord; UCB: umbilical cord blood.

## Discussion

### Summary of Findings

Our systematic review summarizes the data from 12 studies that have assessed the safety, feasibility, and efficacy of allogeneic cell therapy in 153 term and preterm infants worldwide. In addition, it provides the details of 21 ongoing or planned studies using allogeneic cell therapy in 1020 neonates. Most studies used MSCs (134 of 153 infants) derived from UCB,^20,28,29,34,35,40^ umbilical cord tissue,^30,36^ and bone marrow,^32,33^ whereas others used UCB-derived TNCs^31,37^ and hAECs.^39^ The most common indication was bronchopulmonary dysplasia (113 infants). Nine out of 12 studies did not report any serious adverse events related to the therapy and described the therapy as safe and feasible. Other studies reported serious adverse events, such as GVHD (UCB-derived TNCs for Krabbe disease) in 5 infants^31,37,38^ and transient cardiorespiratory compromise (hAECs) in one infant.^39^ The data on efficacy outcomes from comparative studies were similar except for reduced severe BPD with allogeneic cell therapy in 2 studies.^20,35^

### Study Implications

Our systematic review highlights some evidence of the safety and feasibility of allogeneic cell therapy in the neonatal period. However, the findings should be interpreted carefully as data are derived from a few studies evaluating cell therapy in only 153 neonates. Moreover, the cells used in the studies predominantly consist of MSCs (134 of 153 infants) with the remaining being UCB-TNCs and hAECs; hence, these findings should not be generalized to other allogeneic cells. In addition, it is crucial to acknowledge that while MSCs and hAECs were administered as specific cell types, UCB-TNCs involve a scenario where multiple cell types are present. Furthermore, it is important to note that GVHD occurred exclusively with UCB-TNCs, despite prior myeloablative conditioning, highlighting the potential variability of immunologic reactions depending on the specific type of allogeneic cells administered. This finding emphasizes the need for accurate description and characterization of the immunologic reactions based on the cell types employed.

Although MSCs were the predominant cell type studied, their usage has exhibited heterogeneity in terms of cell source (umbilical cord tissue, UCB, and BM) and indications (BPD, IVH, PAIS, and HIE). In addition, it is acknowledged that the efficacy studies conducted on MSCs have been limited in scope, with 2 studies reporting a reduction in the severity of BPD with allogeneic cells. But, these differences were found in the subgroup and exploratory analyses and should be interpreted cautiously.^20,35^ Finally, it is important to note that both published and ongoing research on allogeneic cell therapy is focused on preterm lung disease, with few studies focusing on other relevant conditions, such as brain injury. Hence, further research is warranted to establish and replicate the safety and efficacy of diverse allogeneic cells in different neonatal conditions, involving a larger cohort of neonates.

### Comparison with Previous Research

To date, this is the first systematic review to scrutinize evidence specifically on allogeneic cell therapy in the neonatal period. The study is in keeping with included studies using allogeneic MSCs that demonstrate the safety and feasibility of MSCs in neonates.^20,28-30,32-36,40^ Similar to our study, a recent systematic review by Paton et al focusing on allogeneic UCB-derived TNCs or mononuclear cell therapy in children and adults reported no safety concerns or GVHD.^64^ In contrast, our study identified GVHD in 4 out of 7 patients in one study using UCB-derived TNCs.^37^ It is important to note that all these patients received myeloablative conditioning, and the purpose of cell therapy was transplantation for Krabbe disease. Whereas the indications for allogeneic cell therapy in the review by Paton et al were neurological conditions such as autism, cerebral palsy, stroke, and traumatic brain injury, 6 of 10 studies did not use immunosuppressive prophylaxis.^64^ Therefore, it is important to study the safety of these cell types in the neonatal age group in different settings and determine the true risk of GVHD in this cohort.

### Complexities in Allogeneic Cell Therapy Research

All except 2 studies included no controls. The 2 studies with a control population were small and were not powered to detect meaningful differences in clinical outcomes.^20,35^ But, we acknowledge that well-designed small trials may be required to assess and ensure safety before carrying out large, well-powered clinical trials for therapeutic efficacy. In addition, we recognize that even small trials in this field are very challenging, as they require significant effort in arranging the therapy, including processing the blood, relevant HLA-matching, storing and transporting cells, complying with infection control measures and governance policies, and adhering to the highest standards, as is the case for any stem cell transplantation. Furthermore, significant funding is also required to carry out these trials, which is often challenging. Our search has determined that there are 21 ongoing studies for the use of allogeneic cells for neonatal morbidities, with dates indicating that many planned studies are slow to begin and may not reach completion. For example, an RCT investigating neural progenitor cells and paracrine factors in 120 HIE infants has been underway since 2013 and is yet to be completed.^42^ The trial was registered on the clinicaltrials.gov website in 2016, with no updates since then.

### Strengths and Limitations

Our systematic review has several strengths. It followed the PRISMA reporting guideline and systematically summarized the evidence from published research on allogeneic cell therapy in term and preterm neonates. The search was comprehensive and included several databases and clinical trial registries. The systematic review also highlighted ongoing research and identified critical gaps and the need for further studies. Nonetheless, limitations must be acknowledged. The limitations largely arise from the design of the studies included in this systematic review. Included studies were few, had enrolled a small cohort of patients, had methodological limitations, were primarily single-arm studies, and lacked long-term outcome data. Furthermore, the safety and feasibility of other allogeneic cells are limited, as MSCs were predominantly used. In addition, as in any other cell therapy research, it is challenging to make distinct conclusions as different cells were used for different conditions with different dosing and timing. Finally, no meta-analysis was conducted due to a lack of homogeneous data from 2 or more studies.

## Conclusions

Results from this systematic review summarize the current evidence on the safety, feasibility and efficacy of allogeneic cell therapy in neonates. This primarily involved studies examining the administration of MSCs from different sources, highlighting some evidence regarding the safety and feasibility. However, the review notes that data on efficacy outcomes are very limited, in both the short and long term. Therefore, further research on a broader population of allogeneic cells is required, particularly emphasizing their efficacy related to the predominant morbidities that require therapeutic intervention in the neonatal period.

## Supplementary Material

szad048_suppl_Supplementary_Material

## Data Availability

The data underlying this article will be shared on reasonable request to the corresponding author.
