# Supplementary material for: Allogeneic Cell Therapy Applications in Neonates: A Systematic Review
Source: Stem Cells Transl Med. 2023 Aug 21;12(10):651–64. doi: 10.1093/stcltm/szad048 (PMC10552935; doi:10.1093/stcltm/szad048)
Supplement: szad048_suppl_Supplementary_Material [file szad048_suppl_supplementary_material.docx]

**Supplemental Content**

**eAppendix 1.** Search Strategy

**eTable 1:** Quality assessment of case reports, case series, and studies with no comparator group

**eTable 2:** Quality assessment of non-randomised studies

**eTable 3**: Quality assessment of Randomised Trials

**eAppendix 1.** Search Strategy

**Database:** Embase Classic+Embase <1947 to 2022 October 05>

1 neonate.mp. 50973

2 newborn.mp. or newborn/ 799473

3 neonatal.mp. 335872

4 infant.mp. or infant/ 932851

5 preterm.mp. 123894

6 premature.mp. 250477

7 prematurity/ or prematurity.mp. 147136

8 1 or 2 or 3 or 4 or 5 or 6 or 7 1730880

9 allogeneic.mp. 127513

10 stem cells.mp. or stem cell/ 379114

11 9 or 10 487875

12 8 and 11 19637

13 limit 12 to (human and infant <to one year>) 661

**Database:** Ovid MEDLINE(R) and Epub Ahead of Print, In-Process, In-Data-Review & Other Non-Indexed Citations, Daily and Versions <1946 to October 05, 2022>

1 neonate.mp. 33976

2 newborn.mp. 808508

3 neonatal.mp. 255857

4 preterm.mp. 87959

5 premature.mp. 215309

6 prematurity.mp. 27542

7 allogeneic.mp. or Allogeneic Cells/ 69283

8 1 or 2 or 3 or 4 or 5 or 6 1018650

9 7 and 8 1769

10 limit 9 to (humans and "newborn infant (birth to 1 month)") 911

**Databases**: EBM Reviews - Cochrane Central Register of Controlled Trials <September 2022> and EBM Reviews - Cochrane Database of Systematic Reviews <2005 to October 5, 2022>

1 neonate.mp. [mp=ti, ot, ab, fx, sh, hw, kw, tx, ct] 2364

2 newborn.mp. [mp=ti, ot, ab, fx, sh, hw, kw, tx, ct] 31507

3 neonatal.mp. [mp=ti, ot, ab, fx, sh, hw, kw, tx, ct] 22261

4 preterm.mp. [mp=ti, ot, ab, fx, sh, hw, kw, tx, ct] 16703

5 premature.mp. [mp=ti, ot, ab, fx, sh, hw, kw, tx, ct] 21060

6 prematurity.mp. [mp=ti, ot, ab, fx, sh, hw, kw, tx, ct] 7761

7 infant.mp. [mp=ti, ot, ab, fx, sh, hw, kw, tx, ct] 58730

8 1 or 2 or 3 or 4 or 5 or 6 or 7 88602

9 allogeneic.mp. [mp=ti, ot, ab, fx, sh, hw, kw, tx, ct] 6744

10 stem cells.mp. [mp=ti, ot, ab, fx, sh, hw, kw, tx, ct] 5103

11 9 or 10 11067

12 8 and 11 466

| **Trial Registries** | **Search Strategy and date** | **Articles retrieved** |
| --- | --- | --- |
| Australian New Zealand Clinical Trials Registry (<https://anzctr.org.au/TrialSearch.aspx>) | (allogeneic OR "stem cells") AND (neonate OR neonatal OR newborn OR preterm OR premature OR infant)  Date: 09 Oct 2022 | 11 |
| Chinese Clinical Trial Registry (<http://www.chictr.org.cn/searchprojen.aspx>) | Allogeneic  Date: 09 Oct 2022 | 13 |
| European Clinical Trials Register (<https://www.clinicaltrialsregister.eu/ctr-search/search>) | (allogeneic OR "stem cells") AND (neonate OR neonatal OR newborn OR preterm OR premature OR infant) Filter: Under 18 years  Date: 09 Oct 2022 | 26 |
| International Clinical Trials Registry Platform (<https://trialsearch.who.int/>) | (allogeneic OR "stem cells") AND (neonate OR neonatal OR newborn OR preterm OR premature OR infant)  Date: 09 Oct 2022 | 51 |
| International Standard Randomised Controlled Trial Number Registry  (<https://www.isrctn.com>) | (allogeneic OR "stem cells") AND (neonate OR neonatal OR newborn OR preterm OR premature OR infant)  Date: 09 Oct 2022 | 11 |
| U.S. National Library of Medicine (<https://clinicaltrials.gov/>) | (allogeneic OR "stem cells") AND (neonate OR neonatal OR newborn OR preterm OR premature OR infant)  Filter: child (birth-17)  Filter: Recruitment NOT “suspended” OR “terminated”  Date: 09 Oct 2022 | 239 |

**e-Table 1: Quality assessment of case reports, case series, and studies with no comparator group**

| Domains | Leading explanatory questions | Ahn et al, 2018[1] | Akduman et al, 2021[2] | Allewelt et al, 2018[3] and Escolar et al, 2005[4] | Alvarez-Fuente et al, 2018[5] | Baak et al, 2022[6] | Bozkaya et al, 2022[7] | Cotten et al, 2020[8] | Lesnik et al, 2001[9] | Lim et al, 2018[10] | Powell et al, 2019[11] |
| --- | --- | --- | --- | --- | --- | --- | --- | --- | --- | --- | --- |
| Selection | 1. Does the patient(s) represent(s) the whole experience of the investigator (centre) or is the selection method unclear to the extent that other patients with similar presentation may not have been reported? | Yes | N/A – case report involving 1 patient | Yes | N/A - case series involving 2 patients | Yes | N/A - case report involving 1 patient | Unclear ( 6 patients enroledd, no full-text available to ascertain selection bias) | N/A - case report involving 1 patient | Yes | Yes |
| Ascertainment | 2. Was the exposure adequately ascertained? | Yes | Yes | Yes | Yes | Yes | Yes | Yes | Yes | Yes | Yes |
|  | 3. Was the outcome adequately ascertained? | Yes | Yes | Yes | Yes | Yes | Yes | Yes | Yes | Yes | Yes |
| Causality | 4. Were other alternative causes that may explain the observation ruled out? | Yes (no deaths related to MSC, one infant had seizure which was attributed to sepsis that occured later | No - authors mention that surgery could have led to increased intestinal perfusion and this explanation is not ruled out | Yes - authors provide details of co-interventions r (busulfan, cyclophosphamide, cyclosporine, steroids) but unclear to assess the impact of these therapies. They also mention deaths were related to transplantation. | No - authors unable to rule out potential effect of concomitant steroid administration on outcomes of patient 1 | Yes (fever in one case was attributed to stem cells) | No - authors did not rule out potential effect of other interventions received (phenobarbital, levetiracetam; also possibility of spontaneous recovery) | Yes | Yes | Yes | Yes |
|  | 5. Was there a challenge/rechallenge phenomenon? | N/A | N/A - infant only received one MSC injection | N/A | No - for outcomes measured before & after each dose, no clear pattern of change following each dose | No - infants only received MSCs once | No - infants only received intraventricular & intravenous MSC once | N/A | N/A, it was a matched transplant | N/A - infants only received one hAECs injection | N/A - infants only received MSCs once |
|  | 6. Was there a dose–response effect? | N/A | N/A - only one dose given | N/A | No - no improvement in outcomes with higher doses | No - only one dose given | No - only one dose given | N/A (two similar doses were given) | N/A, it was a matched transplant | N/A - only one dose given | No - no significant difference in outcomes between 2 dosage groups |
|  | 7. Was follow-up long enough for outcomes to occur? | Yes | Yes | Yes | Yes | Yes | Yes | Yes | Yes | Yes | Yes |
| Reporting | 8. Is the case(s) described with sufficient details to allow other investigators to replicate the research or to allow practitioners make inferences related to their own practice? | Yes | Yes | Yes | Yes | Yes | No - insufficient detail of all interventions received by infant and other outcomes following MSC administration | Yes | Yes | Yes | Yes |
| ROB | Overall | 6/8 (Low) | 4/8 (Moderate) | 6/8 (Low) | 4/8 ( Moderate) | 6/8 (Low) | 3/8 (High) | N/A | 5/8 (Moderate) | 6/8 (Low) | 6/8  (Low) |

Note:

hAECs=human amnion epithelial cells

MSCs=mesenchymal stem cells

N/A=not applicable

**e-Table 2: Quality assessment of non-randomised studies**

| Domain | Leading explanatory questions | Chang et al, 2014[12] and Ahn et al, 2017[13] |
| --- | --- | --- |
| Selection | Representativeness of cohort (truly or somewhat representative of average neonatal population) | Yes |
|  | Selection of non-exposed amongst cohort (i.e. sample drawn from same neonatal population) | Yes |
|  | Ascertainment of exposure (secure record) | Yes |
|  | Outcome does not present at start of study | Yes |
| Comparability | Study controls for GA and Sex | Yes (gestational age and birthweight) |
|  | Study controls for additional factors | Yes (Respiratory severity score) |
| Outcome | Assessment of outcome (independent blind) | No - no mention of independent or blind outcome assessment.  Follow-up outcomes were assessed through standardized interviews, with no mention of independent or blind assessment |
|  | Was follow-up complete (adequate follow-up period for event to occur) | Yes |
|  | Adequacy of follow-up of cohorts (>70% followed up) | Yes |
| Overall risk |  | Low (8/9) |

**e-Table 3: Quality assessment of Randomised Trials**

| Risk of bias domain | Ahn et al, 2021[14] |
| --- | --- |
| Risk of bias arising from the randomization process | Low - no mention of allocation concealment however baseline differences across groups, including subgroups, are not significant |
| Risk of bias due to deviations from the intended interventions | Low |
| Risk of bias due to missing outcome data | Low |
| Risk of bias in measurement of the outcome | Low |
| Risk of bias in selection of the reported result | Low |

**References for Supplemental Content:**

1. Ahn SY, Chang YS, Sung SI, Park WS: Mesenchymal Stem Cells for Severe Intraventricular Hemorrhage in Preterm Infants: Phase I Dose-Escalation Clinical Trial. *Stem Cells Transl Med* 2018, 7(12):847-856.

2. Akduman H, Dilli D, Ergun E, Cakmakci E, Celebi SK, Citli R, Zenciroglu A: Successful Mesenchymal Stem Cell Application in Supraventricular Tachycardia-Related Necrotizing Enterocolitis: A Case Report. *Fetal Pediatr Pathol* 2021, 40(3):250-255.

3. Allewelt H, Taskindoust M, Troy J, Page K, Wood S, Parikh S, Prasad VK, Kurtzberg J: Long-Term Functional Outcomes after Hematopoietic Stem Cell Transplant for Early Infantile Krabbe Disease. *Biol Blood Marrow Transplant* 2018, 24(11):2233-2238.

4. Escolar ML, Poe MD, Provenzale JM, Richards KC, Allison J, Wood S, Wenger DA, Pietryga D, Wall D, Champagne M *et al*: Transplantation of umbilical-cord blood in babies with infantile Krabbe's disease. *N Engl J Med* 2005, 352(20):2069-2081.

5. Alvarez-Fuente M, Arruza L, Lopez-Ortego P, Moreno L, Ramirez-Orellana M, Labrandero C, Gonzalez A, Melen G, Cerro MJD: Off-label mesenchymal stromal cell treatment in two infants with severe bronchopulmonary dysplasia: clinical course and biomarkers profile. *Cytotherapy* 2018, 20(11):1337-1344.

6. Baak LM, Wagenaar N, van der Aa NE, Groenendaal F, Dudink J, Tataranno ML, Mahamuud U, Verhage CH, Eijsermans R, Smit LS *et al*: Feasibility and safety of intranasally administered mesenchymal stromal cells after perinatal arterial ischaemic stroke in the Netherlands (PASSIoN): a first-in-human, open-label intervention study. *Lancet Neurol* 2022, 21(6):528-536.

7. Bozkaya D, Ceran B, Ozmen E, Okman E, Dizdar EA, Oguz SS, Bozkaya IO: A New Hope in the Treatment of Intraventricular Haemorrhage in Preterm Infants: Mesenchymal Stem Cells. *Turk Neurosurg* 2022, 32(2):344-346.

8. Cotten C, Fisher K, Kurtzberg J, Simmons R: Phase I trial of allogeneic umbilical cord tissue-derived mesenchymal stromal cells in neonates with hypoxic-ischemic encephalopathy. *Cytotherapy* 2020, 22(5):S192.

9. Lesnik JJ, Singh GK, Balfour IC, Wall DA: Steroid-induced hypertrophic cardiomyopathy following stem cell transplantation in a neonate: a case report. *Bone Marrow Transplant* 2001, 27(10):1105-1108.

10. Lim R, Malhotra A, Tan J, Chan ST, Lau S, Zhu D, Mockler JC, Wallace EM: First-In-Human Administration of Allogeneic Amnion Cells in Premature Infants With Bronchopulmonary Dysplasia: A Safety Study. *Stem Cells Transl Med* 2018, 7(9):628-635.

11. Powell SB, Silvestri JM: Safety of Intratracheal Administration of Human Umbilical Cord Blood Derived Mesenchymal Stromal Cells in Extremely Low Birth Weight Preterm Infants. *J Pediatr* 2019, 210:209-213 e202.

12. Chang YS, Ahn SY, Yoo HS, Sung SI, Choi SJ, Oh WI, Park WS: Mesenchymal stem cells for bronchopulmonary dysplasia: phase 1 dose-escalation clinical trial. *J Pediatr* 2014, 164(5):966-972 e966.

13. Ahn SY, Chang YS, Kim JH, Sung SI, Park WS: Two-Year Follow-Up Outcomes of Premature Infants Enrolled in the Phase I Trial of Mesenchymal Stem Cells Transplantation for Bronchopulmonary Dysplasia. *J Pediatr* 2017, 185:49-54 e42.

14. Ahn SY, Chang YS, Lee MH, Sung SI, Lee BS, Kim KS, Kim AR, Park WS: Stem cells for bronchopulmonary dysplasia in preterm infants: A randomized controlled phase II trial. *Stem Cells Transl Med* 2021, 10(8):1129-1137.
